# Mitochondrial donation by stem cells: potential for novel therapeutics

**DOI:** 10.1186/1755-8166-7-S1-I59

**Published:** 2014-01-21

**Authors:** Anurag Agrawal

**Affiliations:** 1 CSIR-Institute of Genomics & Integrative Biology, New Delhi, India

## 

There is increasing interest in whether mesenchymal stem cells (MSC) act as mitochondrial donors for rescuing injured cells. Islam *et al* (Nature Medicine, 2012) showed conclusively, using a mouse model of acute lung injury, that MSC mediated mitochondrial donation can lead to survival benefits (3). Contemporaneous work from Dr. Anurag Agrawal’s lab reveals the molecular regulation of mitochondrial movement during donation and shows how this can be engineered to increase therapeutic efficacy of MSC. They find that Miro1, a calcium sensitive mitochondrial Rho-GTPase that attaches mitochondria to KIF5 motor proteins on microtubules and regulates neuronal mitochondrial trafficking, is necessary for mitochondrial transfer by MSC. Overexpressing Miro1 in MSC (MSCmiro^Hi^) led to increased mitochondrial transfer and therapeutic efficacy in mouse models of lung injury as well as asthma. This is a significant addition to the field of mitochondrial biology and stem cell therapeutics. It is also of general interest to physicians and members of public with interest in regenerative medicine, because this is highly translatable into more effective therapies.

